# Artificial intelligence for volumetric measurement of cerebral white matter hyperintensities on thick-slice fluid-attenuated inversion recovery (FLAIR) magnetic resonance images from multiple centers

**DOI:** 10.1038/s41598-024-60789-x

**Published:** 2024-05-02

**Authors:** Masashi Kuwabara, Fusao Ikawa, Shinji Nakazawa, Saori Koshino, Daizo Ishii, Hiroshi Kondo, Takeshi Hara, Yuyo Maeda, Ryo Sato, Taiki Kaneko, Shiyuki Maeyama, Yuki Shimahara, Nobutaka Horie

**Affiliations:** 1 Department of Neurosurgery, Graduate School of Biomedical and Health Sciences, Hiroshima University, 1-2-3 Kasumi, Minami-Ku, Hiroshima, Hiroshima 734-8551 Japan; 2 Department of Neurosurgery, Shimane Prefectural Central Hospital, 4-1-1 Himebara, Izumo, Shimane 693-0068 Japan; 3 LPIXEL Inc, 1-6-1 Otemachi, Chiyoda-Ku, Tokyo, 100-0004 Japan; 4 Department of Radiology, The University of Tokyo Hospital, 7-3-1 Hongo, Bunkyo-Ku, Tokyo, 113-8655 Japan

**Keywords:** White matter hyperintensity, Artificial intelligence, Magnetic resonance imaging, Fluid-attenuated inversion recovery, Cognitive ageing, Software, Nervous system, Magnetic resonance imaging, Neuroscience, Medical research, Neurology, Mathematics and computing

## Abstract

We aimed to develop a new artificial intelligence software that can automatically extract and measure the volume of white matter hyperintensities (WMHs) in head magnetic resonance imaging (MRI) using only thick-slice fluid-attenuated inversion recovery (FLAIR) sequences from multiple centers. We enrolled 1092 participants in Japan, comprising the thick-slice Private Dataset. Based on 207 randomly selected participants, neuroradiologists annotated WMHs using predefined guidelines. The annotated images of participants were divided into training (n = 138) and test (n = 69) datasets. The WMH segmentation model comprised a U-Net ensemble and was trained using the Private Dataset. Two other models were trained for validation using either both thin- and thick-slice MRI datasets or the thin-slice dataset alone. The voxel-wise Dice similarity coefficient (DSC) was used as the evaluation metric. The model trained using only thick-slice MRI showed a DSC of 0.820 for the test dataset, which is comparable to the accuracy of human readers. The model trained with the additional thin-slice dataset showed only a slightly improved DSC of 0.822. This automatic WMH segmentation model comprising a U-Net ensemble trained on a thick-slice FLAIR MRI dataset is a promising new method. Despite some limitations, this model may be applicable in clinical practice.

## Introduction

White matter hyperintensities (WMHs) are common neuroimaging findings characterized by high signals either as periventricular hyperintensities (PVHs) or deep subcortical WMHs (DSWMHs) in fluid-attenuated inversion recovery (FLAIR) sequences of head magnetic resonance imaging (MRI)^1^. Many reports have shown associations of the WMH degree with ischemic stroke, depression, dementia, and emotional disorders^2–8^. Although the WMH prevalence is higher in older individuals, studies of healthy younger people have reported that the WMH degree is also correlated with the execution of speed-demanding functions, that is, subfrontal cortical functions, such as word recall^6–10^.

Brain Dock is a widely implemented brain checkup system that uses MRI and magnetic resonance angiography to detect asymptomatic brain diseases, such as unruptured cerebral aneurysms and WMHs at an early stage, thereby preventing stroke and dementia in healthy individuals in Japan^11,12^. During a Brain Dock, the evaluation of WMHs is required, using a standard database with the aim of accumulating data on the diagnosis and early detection of disease risk^11–13^. To date, manual interpretation by neuroradiologists has been considered an effective method for quantitatively assessing WMHs^14^. However, this approach is not only time-consuming and labor-intensive but also has the disadvantage of high interrater variability (10–68%), which can be expected to result in significant bias, making large-scale studies using quantitative evaluation difficult^14–17^.

Recently, artificial intelligence (AI) has been used for automated reading and volumetric measurements. Although many publications have reported the use of AI to assess brain atrophy on T1-weighted images (T1WIs), reports on AI algorithms for automated WMH segmentation using only FLAIR images are limited^15,18^. The size, number, shape, and location of WMHs are heterogeneous and age-dependent, making them difficult to evaluate using AI. Moreover, WMHs are present in a variety of diseases, requiring background information, such as patient history and disease course, and multiple MRI sequences for differentiation^15,19,20^. Only a few of the reported AI algorithms are applicable in clinical practice because many are limited to specific diseases, have small sample sizes, evaluate multiple sequences, or require a thin slice thickness as the imaging condition^19,21,22^. To ensure applicability in large-sized clinical practice for brain screening, accurate results must be provided consistently under imaging protocols for various WMHs, often requiring multicenter collaborative studies^23,24^. Only a few studies to date have validated the AI algorithm for automatic WMH segmentation by FLAIR alone in a multicenter setting^25^. If AI algorithms for automated WMH segmentation using only FLAIR images were developed and automatically classified in Brain Dock, the scientific contribution to preventive medicine in healthy people would be immeasurable. The initial purpose of this study was to develop a new AI software that can automatically extract and measure WMH volumes on head MR images using only thick-slice FLAIR images from multiple centers, in accordance with the Japan Brain Dock Society standards. In the future, we plan to develop an AI software that can automatically classify WMHs.

## Results

**Figure 1:**
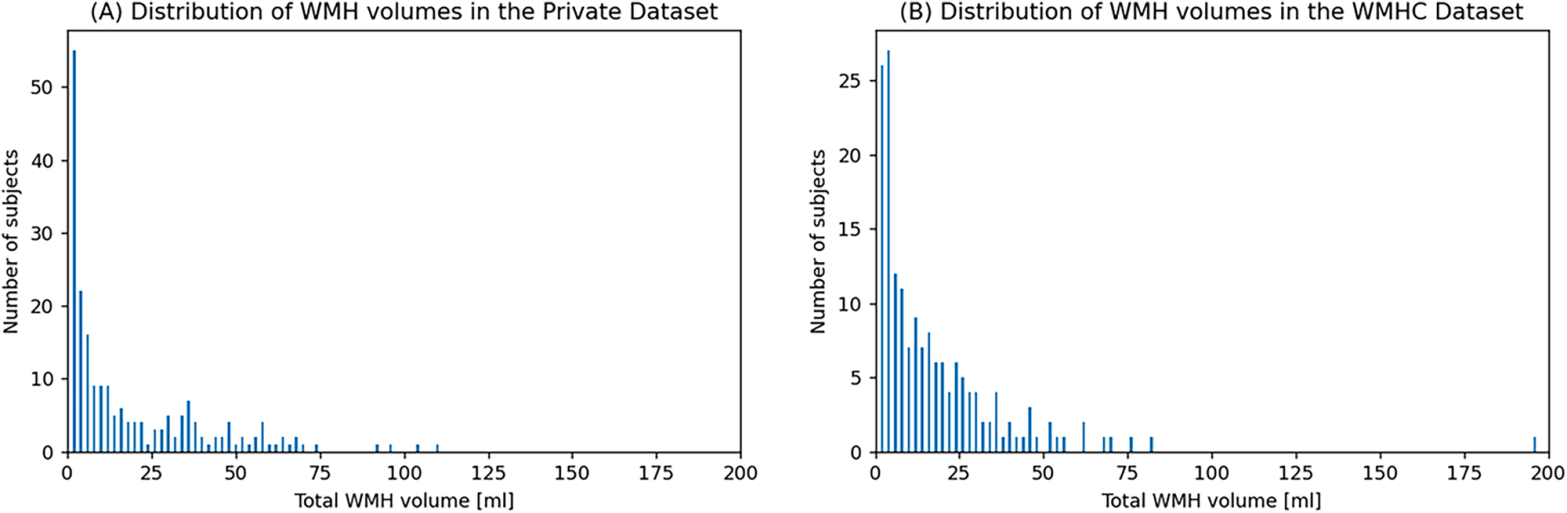
Histograms showing the WMH volume distributions throughout the annotated Private Dataset (n = 207) and WMHC Dataset (n = 170). The WMH volume histograms of the Private Dataset (**a**) and WMHC Dataset (**b**) are shown. The x-axis represents the total WMH volume in each participant, and the y-axis represents the counts. WMH: white matter hyperintensity, WMHC: white matter hyperintensity segmentation challenge.

According to the five neuroradiologists participating in this study, the average WMH volume was 18.11 ± 22.07 mL, with a maximum of 109.45 mL and a minimum of 0.00 mL indicating the absence of a WMH. The distribution of WMH volumes in the Private Dataset (PR) (Fig. 1a) closely resembled that in the WMH Segmentation Challenge (WMHC) Dataset (Fig. 1b). Given the potential for biased evaluation data to negatively impact generalization performance on unseen data, the PR was expected to evaluate generalization performance with similar accuracy to that of the WMHC Dataset. Moreover, the unannotated images of 885 participants were utilized for pseudo-labeling to enhance model performance.

### Quantitative analysis

Table 1 shows the evaluation results of WMH segmentation based on the Dice similarity coefficient (DSC), Recall, precision, and modified Hausdorff distance (95th percentile of the Hausdorff distance in mm) were compared. The test results for the PR and WMHC Dataset are shown separately. The PR model demonstrated high performance with a DSC of 0.8 or higher on the PR, whereas its performance on the WMHC Dataset was not as high. The PR + WMHC model, which included the WMHC Dataset for training, showed improved performance and the ability to handle both thick- and thin-slice MRI datasets simultaneously. However, its performance on the PR improved only marginally, with a DSC increase of only 0.002. This modest degree of improvement might be attributed to domain differences between these datasets, suggesting that the thin-slice dataset does not significantly enhance performance on the thick-slice dataset. Additionally, the model trained only on the WMHC Dataset exhibited overfitting to the test dataset.

**Table 1:** Assessment of WMH segmentation by the U-Net ensemble models.

Model name	Training dataset	Private Dataset for the test dataset (n = 69)				WMHC Dataset for the test dataset (n = 110)			
		DSC	Recall	Precision	H95	DSC	Recall	Precision	H95
PR	Private Dataset	0.820	0.788	0.855	14.50	0.742	0.862	0.653	22.78
PR + WMHC	Private and WMHC Datasets	0.822	0.789	0.858	14.92	0.826	0.877	0.781	7.97
WMHC	WMHC Dataset	0.676	0.531	0.931	16.20	0.833	0.874	0.796	5.61

DSC: Dice similarity coefficient, H95: modified Hausdorff distance (95th percentile [mm]), PR: Private Dataset, WMH: white matter hyperintensity, WMHC: white matter hyperintensity segmentation challenge.

Regarding the performance of the existing commercial software that served as the basis for the algorithm in this study, it had a DSC of 0.635 for the PR, whereas the new algorithm showed a DSC of 0.820.

### Qualitative analysis

**Figure 2:**
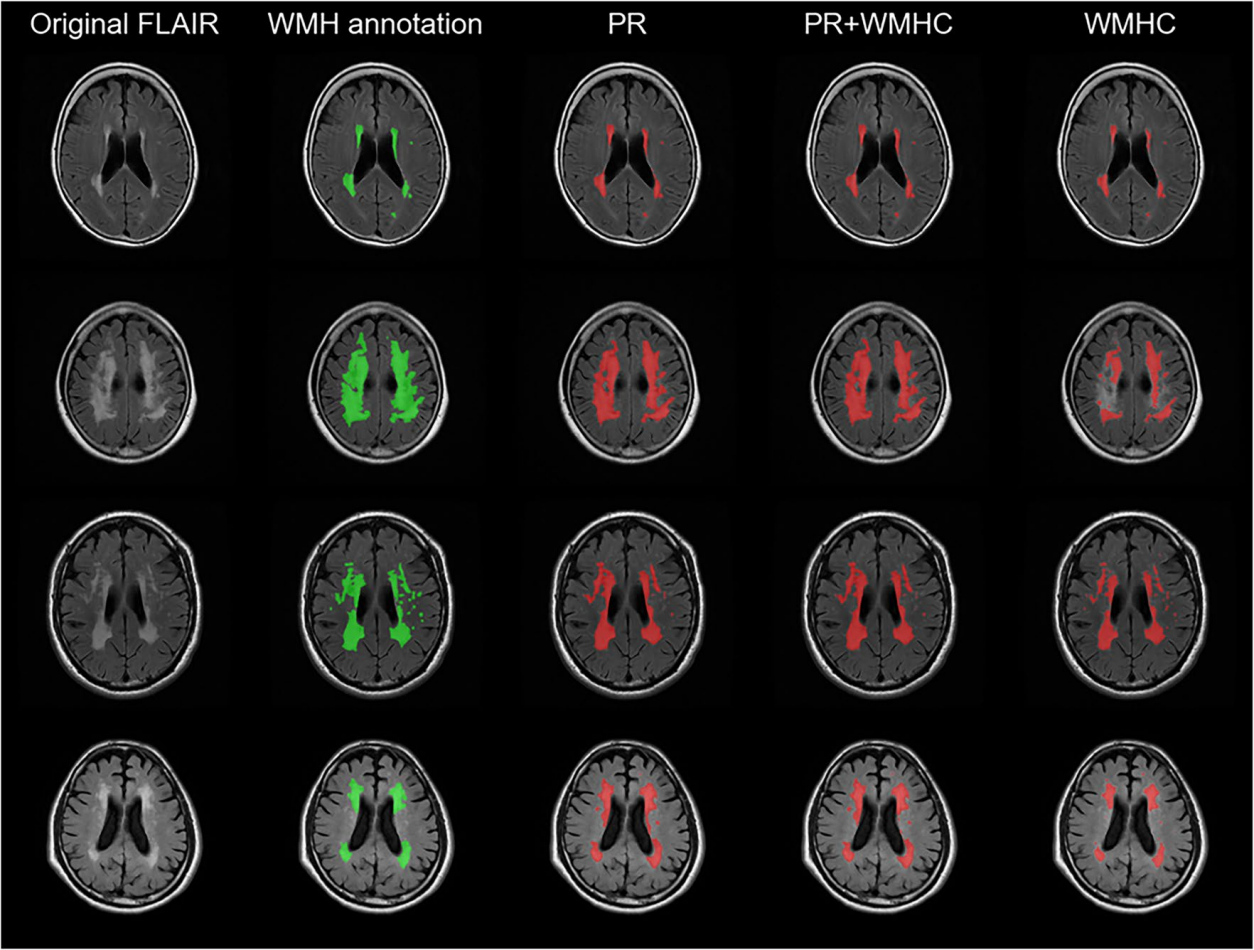
Segmentation results for Private Dataset and WMHC Dataset models used with the test dataset. The columns from left to right show the original FLAIR image, ground-truth WMH annotations, and segmentation results for the PR, PR + WMHC, and WMHC models. The first row shows a TP case in which all models correctly detected WMHs, the second shows FN regions in the WMHC results, and the third row shows small FN regions in the PR and PR + WMHC results. The last row shows the difference in FP regions between PR and PR + WMHC results. FLAIR: fluid-attenuated inversion recovery, FN: false negative, FP: false positive, PR: Private Dataset, TP: true positive, WMH: white matter hyperintensity, WMHC: WMH Segmentation Challenge.

Figure 2 shows the segmentation results of the PR and WMHC Dataset models used with the test dataset. The top row presents a participant with a relatively small WMH volume. All models successfully detected regions that were similar to the ground truth. In a participant with extensive WMHs, as in the second row, both the PR and PR + WMHC models accurately detected the WMH regions, whereas the WMHC model exhibited false negatives (FNs), leading to a lower DSC. However, in a participant with small punctate WMHs, as observed in the right hemisphere in the third row, the WMHC model demonstrated better results compared with the other two models. The differences between the PR and the PR + WMHC models are shown in the last row. False positives (FPs), which occurred in the PR model, were reduced in the PR + WMHC model. For all other participants, the difference between these two models was minimal, which resulted in a slight DSC difference of 0.002.

A tendency toward FPs and FNs was observed in the PR model in areas with small punctate WMHs, near the lateral ventricles, near the septum pellucidum, lacunar infarcts, or where the boundary between WMHs and other structures was ambiguous. As shown in Fig. 2, row 3, the model missed the small punctate WMHs in the left hemisphere. Conversely, in the example from Fig. 2, row 4, punctate FPs can be observed in the left hemisphere. The figure also shows FPs near the surface of the lateral ventricle body, a region where the boundary between a WMH and other structures might be indistinct. In the same example, the FPs were only observed in the PR and PR + WMHC models. For such cases with ambiguous boundaries, the PR model tends to detect slightly excessively, suggesting the possibility of slight over-annotation in the PR for training. While the examples in Fig. 2, rows 1 and 2, are generally accurate, an unintended “hole” appeared in the right hemisphere of Fig. 2, row 2. Furthermore, the recall of the PR model was lower than its precision, indicating that the model tended to have an under-detection bias on the PR.

**Figure 3:**
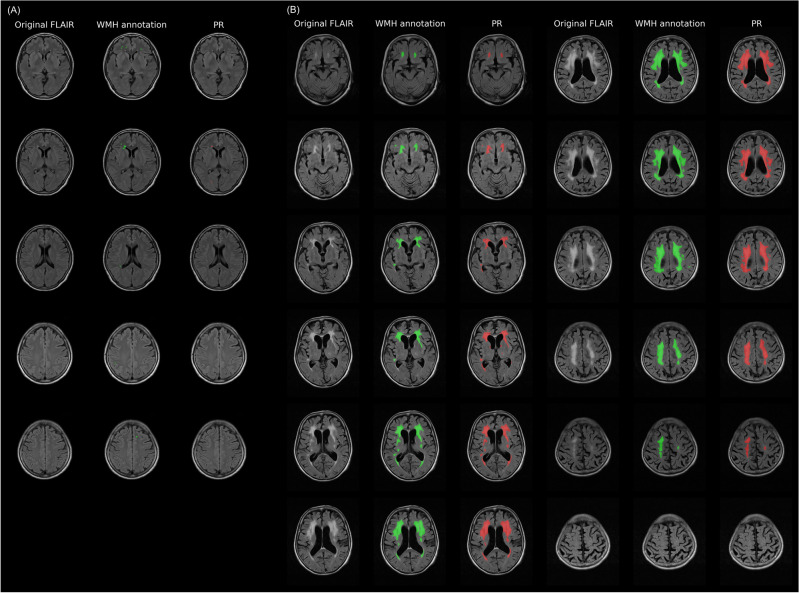
Segmentation results of the PR model for cases with the lowest and highest DSC values from the Private Dataset. (**a**) The case with the lowest DSC value of 0.158; (**b**) the case with the highest DSC value of 0.901. The columns titled “Original FLAIR,” “WMH annotation,” and “PR” display the original FLAIR images; the ground-truth WMH annotations and the segmentation results of the PR model are shown in green and red, respectively. DSC: Dice similarity coefficient, FLAIR: fluid-attenuated inversion recovery, PR: Private Dataset, WMH: white matter hyperintensity.

To better understand variations on a case-by-case basis, the DSC for each case was calculated using the PR model results of the PR. The DSC values ranged from a minimum of 0.158 to a maximum of 0.901. Figure 3a,b show the cases with the lowest and highest DSCs, respectively. The case with the lowest DSC, at 0.158, missed small punctate WMHs, and the missed area accounted for a large portion of the total WMH area. Conversely, the case with the highest DSC (0.901) showed a larger total WMH area. Although some FNs or FPs were observed, their proportions to the total count were limited, resulting in a high DSC.

### Processing time analysis

Processing time measurements were conducted for thick-slice MR images, revealing an average processing time of 18.5 s per case.

## Discussion

In this study, we evaluated the performance of an AI using automatically extracted volumetric measurement of WMH lesions on head MR images on thick-slice FLAIR images. The results showed that the DSC in our study reached 0.820, which is comparable to human reading accuracy.

Although various algorithms for AI-based automated volumetric measurements of WMHs have been reported in recent years, very few are applicable in clinical practice^19,21,22^. This is because most previous studies on AI and volumetric measurements of WMHs, many were limited to specific diseases, such as Alzheimer’s disease or multiple sclerosis, and had relatively small sample sizes of approximately 100 patients^26–28^ and an algorithm that used three-dimensional (3D) networks with thin-slice image data with 1–3 mm slice thicknesses^27,29,30^. Indeed, thin-slice MRI is reportedly superior to thick-slice MRI in detecting lesions in small areas, such as the brainstem, and in segmentation accuracy^31^. Thick-slice MRI, as used in this study, may reduce the accuracy of volumetric measurements in small lesions. However, thin-slice MRI is only widely applied in some developed countries and is not currently commonly used worldwide and in developing countries; MRI with thick slices of approximately 5 mm, as used in this study, remains the most commonly applied method for routine work^14^. Because thick-slice data is the mainstream in Brain Dock and we aimed to develop a calculation method based on existing Brain Dock data and the default imaging method, we used thick-slice data in this study. The size, number, shape, and location of WMHs are heterogeneous and age-dependent, making reliable and objective assessments difficult^14,24^. In the future, if thin-slice FLAIR imaging becomes the mainstream for brain screening, additional considerations may be necessary.

WMHs result primarily from aging processes, such as demyelination and axonal loss, both of which occur as a result of small vessel disease of the brain^7^. Thus, WMHs are frequently observed in older asymptomatic patients, although they can also occur in a variety of other diseases, including demyelinating diseases such as multiple sclerosis; genetic diseases such as cerebral autosomal dominant arteriopathy with subcortical infarcts and leukoencephalopathy; infectious diseases such as human immunodeficiency virus encephalopathy; psychiatric disorders such as depressive disorders and schizophrenia; and various other diseases such as autoimmune diseases, neoplastic diseases, intoxication, and hypoxic encephalopathies^32^. Therefore, volumetric detection of WMHs in younger people is an important aspect of brain checkups, and lifestyle improvements are needed in examinees with increased WMH volume over time.

This study proposes a new AI software that solves these problems, and its major feature is the analysis of 5-mm thick-slice FLAIR images. Moreover, this study evaluated both the PR and the WMHC Dataset as thick (5 mm) and thin (1–3 mm) slices, respectively, with a sample size of over 200 participants. The results showed that the WMH segmentation accuracy based on two-dimensional (2D) FLAIR images with 5 mm slices, which is widely used in general hospitals, was comparable to that of previous studies. This suggests that the proposed AI algorithm can be applied in clinical practice.

We only identified 39 publications that analyzed the automatic segmentation of WMHs on head MRI using AI similar to the procedure in the present study. Of these, we reviewed 17 publications since 2018, including the present study (Table 2). The sample size varied from 20 to 1092 overall, with the sample size of this study being the largest. Multiple MRI sequences analyzing both T1WI and FLAIR images were identified in 12 studies^20,22,26,33–38^, and only one study in addition to the present study evaluated only FLAIR images. Regarding the MRI field strength, nearly all studies, including ours, used 3 T MRI. Regarding the segmentation accuracy, the DSC values ranged from 0.41 to 0.83, showing a wide range of accuracy. The DSC of the AI model in this study was 0.820, indicating higher accuracy compared with that of previous studies overall. Therefore, the model developed in the present study demonstrably has a better performance than that of existing AI models.

**Table 2:** Review of recent studies on the automatic segmentation of cerebral white matter hyperintensities in head MRI using AI.

Author/year	Sample size	Breakdown of samples	MRI		Type of segmentation	Average spatial agreement with reference segmentation
			Sequence	Field strength (T)		
Manjón JV/2018	128	N/A	T1WI, FLAIR	1.5, 3	Deep learning	DSC: 0.78
Knight J/2018	96	N/A	FLAIR	1.5, 3	Supervised	DSC: 0.41–0.70
Qin C/2018	88	N/A	T1WI, FLAIR	3	Deep learning	DSC: 0.67
Ling Y/2018	156	N/A	T1WI, FLAIR	1.5, 3	Supervised	DSC: 0.76
Park BY/2018	148	N/A	T1WI, FLAIR	1.5	Supervised	DSC: 0.65–0.67
Atlason HE/2019	170	Train: 60, test: 110	T1WI, T2WI, FLAIR	1.5, 3	Unsupervised	DSC: 0.53–0.67, TPR: 0.25–0.40
Sundaresan V/2019	133	N/A	T1WI, T2WI, FLAIR	1.5, 3	Supervised	DSC: 0.77, TPR: 0.73–0.98
Wu D/2019	135	Train: 15, test: 120	T1WI, FLAIR	3	Supervised	DSC: 0.62, FPR: 0.35, FNR: 0.37
Wu J/2019	60	N/A	T1WI, FLAIR	1.5, 3	Deep learning	DSC: 0.78
Ding T/2020	20	Train: 15, test: 5	T1WI, FLAIR	3	Supervised	DSC: 0.78, TPR: 0.70
Fiford CM/2020	80	Train: 20, test: 60	T1WI, FLAIR	3	Unsupervised	DSC: 0.74
Hong J/2020	148	N/A	T1WI, FLAIR	3	Deep learning	TPR: 0.87, FDR: 0.10
Liu L/2020	60	Test: 60	T1WI, FLAIR	1.5, 3	Deep learning	DSC: 0.83
Rachmadi MF/2020	60	Test: 60	T1WI, T2WI, FLAIR	3	Unsupervised	DSC: 0.47–0.56, TPR: 0.47
Tran P/2022	60	N/A	T1WI, FLAIR	N/A	N/A	DSC: 0.67
Zhu W/2022	1,045	Cross-validation: 849	T1WI, FLAIR	1.5, 3	Supervised	DSC: 0.792
Present study/2023	1,092	Train: 138 + 885, test: 69	FLAIR	1.5, 3	Supervised	DSC: 0.820

AI: artificial intelligence, DSC: dice score coefficient, FDR: false discovery rate, FLAIR: fluid-attenuated inversion recovery, FNR: false negative rate, FPR: false positive rate, MRI: magnetic resonance imaging, N/A: not applicable, T: Tesla, T1WI: T1-weighted imaging, T2WI: T2-weighted imaging, TPR: true positive rate.

The unique feature of our study is that we have developed an AI that performs better than previous AI models using “thick slices” and “FLAIR images only” in accordance with the standards of the Japan Brain Dock Society. Notably, the performance of this AI model was tested on the largest sample size in a study of this type of more than 1000 cases, using data from multiple institutions. In addition, the processing time when performing segmentation was as fast as 18.5 s per case for our AI model. When corrected for comparison with a previous study, the equivalent value was approximately 1.8 min. This is faster than the approximate processing time of 5 min using a supervised deep learning-based method reported in the previous study^32^. Therefore, this AI algorithm seems more appropriate for real-world clinical adaptation. MRI with thick slices and FLAIR imaging are only simple methods for brain checkups but have an advantage in that MRI examinations are easily accessible for patients and versatile. Our findings can also be used as a basis for PVH and DSWMH grading as proposed by the Japan Brain Dock Society. The ultimate goal of our AI model is to classify PVH and DSWMH grading to screen normal participants at risk of early-onset dementia to prevent or delay the onset of dementia through lifestyle guidance.

This study had several limitations. First, approximately 10 physicians diagnosed the images for annotation, possibly leading to individual differences in physician judgment, resulting in diagnostic bias. However, all physicians in this study were board-certified radiologists from the Japan Radiological Society or neuroradiologists with more than 7 years of experience and board-certified neurosurgeons from the Japan Neurosurgical Society; therefore, the diagnostic results should be of a uniformly high standard. Second, because only high-intensity areas on FLAIR images were evaluated in this study, lacunar infarction might not have been clearly distinguished because only two lacunar infarctions were included in the training dataset. We would like to develop another AI to distinguish between WMHs and lacunar infarctions after acquiring a lacunar infarctions dataset in the near future. Third, a detailed analysis differentiating between PVH and DSWMH within WMHs was not conducted in this study; however, our research is currently ongoing. Fourth, although the data in this study was normalized by scaling the data to the range of 0.0–1.0, some bright artifacts might have affected the scaled value of the lesion. Although various methods are used for normalization, we used a simple normalization method and managed the intensity variations through contrast augmentation. Other approaches, such as ensuring uniform contrast during the normalization stage, were not validated in this study. Finally, this study was based only on FLAIR images for our purpose, which were not combined with other sequence images such as T1WIs, T2WIs, diffusion-weighted images, and other images that are useful for differentiation. Because the most clinically important imaging method is FLAIR for the diagnosis of WMHs, we believe that the development of a new AI software automatically extracting and measuring WMH volumes using only thick-slice FLAIR images is most meaningful for initial brain screening. As the next step, we plan to create an AI algorithm that combines FLAIR images with other sequences such as T1WIs to differentiate lesions and classify WMHs in the near future.

In conclusion, the automatic WMH segmentation model based on a U-Net ensemble trained on a thick-slice FLAIR MRI dataset showed a DSC of 0.820 and a promising AI algorithm. This model may be applicable in clinical practice for brain checkups.

## Methods

### Ethics approval and informed consent

This study was approved by the Institutional Review Board of Hiroshima University (approval number: E2022-0262). All study protocols were developed according to the guidelines of Hiroshima University Hospital. Individual data were anonymized and collected during routine Brain Dock examinations; thus, all participants provided informed consent based on an opt-out method. Moreover, in this study, we were provided with anonymized data; therefore, the authors did not have access to personally identifiable participant information during or after data collection.

### Study design

In this study, a new automatic WMH segmentation model was developed based on existing commercial software (EIRL Brain Metry version 1.13.0, LPIXEL Inc., Tokyo, Japan). This software employs U-Net for segmentation. Although the core usage of U-Net remains consistent, we replaced the backbone network with a new model and also explored the model ensembles.

The WMH segmentation model of the software was updated using a newly collected dataset, the PR. This new model was named “PR” as it was trained using the PR. The model performance was also evaluated using the publicly available WMHC Dataset^29^. The WMHC Dataset has thinner slices than those in the PR.

For validation, two additional models were trained using the same training procedure. One was a model trained using both the PR and the WMHC Dataset (PR + WMHC model) to evaluate the effect of adding an MRI dataset with thin slices to the PR with thick slices. The other was a model trained using only the WMHC Dataset (the WMHC model) to evaluate the performance of a model trained using only a thin-slice MRI dataset.

#### Private dataset

For this dataset, 1092 FLAIR MR images were collected from three clinics in Japan. To minimize potential biases related to the WMH severity, data were gathered in a manner that ensured a balanced distribution of PVH/DSWMH grades (0–4) as determined by each clinic. The WMH annotation process was restricted to 207 randomly selected participants because of the annotation costs. Nearly all the annotated WMHs were age-related, and 2 of these 207 participants had lacunar infarcts. The slice thickness of the annotated data ranged from 5 to 6 mm, with an average of 5.37 mm for the entire dataset. Detailed spatial characteristics, including slice thickness, are presented in Table 3. The data were acquired using nine different types of scanners, and the detailed MRI parameters are listed in Supplementary Table S1.

**Table 3:** Characteristics of the Private Dataset comprising annotated images of 207 participants.

Vendor	Scanner type ID^a^	Scanner model	Magnetic field strength (T)	Acquisition matrix	Slice thickness (mm)	Training	Test
GE Healthcare	1	DISCOVERY MR750w	3	320 × 192	6.0	32	0
(Chicago, IL, USA)	2	Optima MR450w	1.5	224 × 224	5.0	0	2
	3	SIGNA Architect	3	288 × 224	5.0	3	4
	4	SIGNA EXCITE	1.5	288 × 224	6.0	0	6
	5	Signa HDxt	1.5	288 × 224	5.0	0	1
	5	Signa HDxt	1.5	320 × 320	6.0	1	0
	5	Signa HDxt	1.5	288 × 224	6.0	0	1
	5	Signa HDxt	1.5	224 × 192	6.0	37	0
	6	Signa HDxt	3	288 × 192, 224	5.0	6	11
Philips	7	Ingenia	1.5	268 × 246	5.0	19	8
(Amsterdam, the Netherlands)	7	Ingenia	1.5	320 × 223	5.0	1	0
	7	Ingenia	1.5	320 × 198, 242	5.0	8	25
Siemens Healthineers	8	Avanto	1.5	256 × 117	5.0	15	5
(Erlangen, Germany)	9	Symphony	1.5	320 × 211, 230	5.0	16	6

^a^Scanner type ID: a unique ID for each combination of scanner and magnetic field strength; T: Tesla.

Five neuroradiologists shared the annotation efforts of WMHs for the 207 selected participants. To minimize interrater variability and ensure annotation quality, an annotation guideline was developed under the supervision of neuroradiologists. The following guidelines were provided for areas prone to individual variations: (a) the area around the ventricles, sulci, and longitudinal fissure may show a non-WMH signal; therefore, an annotation is only made when a WMH is clearly visible on neighboring upper and lower slices, (b) the septum pellucidum of the lateral ventricles should not be annotated, (c) only WMHs in the cerebrum should be annotated, and (d) lacunar infarcts and enlarged perivascular spaces (EPVSs) should be excluded. The imaging differentiation between lacunar infarcts, EPVSs, and WMHs is that a WMH is depicted as a clear hyperintense lesion on FLAIR, whereas an EPVS is depicted as an iso-to-hypointense lesion and a lacunar infarct is depicted as an iso-to-hyperintense lesion with a hypointense center^11,13^. In addition, WMHs are found mainly in the cerebral white matter, whereas EPVSs and lacunar infarcts are found in the basal ganglia, in addition to the cerebral white matter.

For the model development, the annotated datasets were randomly divided into training (n = 138) and test (n = 69) datasets. To ensure an accurate evaluation, a second annotation review was conducted on 69 test participants. The review was performed by three neuroradiologists who differed from the five physicians employed in the initial annotation process. The reviewers were asked to modify the previously annotated WMH regions if needed. As a result, several modifications were made to the test dataset, thereby ensuring the reliability of the evaluation criteria.

#### WMH segmentation challenge dataset

For the evaluation using an external dataset, the WMHC Dataset was employed^29^. A competition using this dataset was originally held at the 20th International Conference on Medical Image Computing and Computer-Assisted Intervention in 2017 in Quebec City, Quebec, Canada. After the competition, models could be submitted via their website until December 2022. Afterward, the previously undisclosed test dataset was made available. Several previous studies have used this dataset^15,26,31,32^, and our study assessed performance using the same dataset. Although the dataset included both FLAIR images and T1WIs, only FLAIR images were used in this study.

The WMHC Dataset consisted of 60 training and 110 test participants. These participants were sourced from three institutes located in the Netherlands and Singapore. The data were acquired using five different types of scanners. The average slice thickness of the FLAIR sequences in this dataset was 2.23 mm. Notably, the average slice thickness was approximately 41% that of the PR. The WMH volume averaged 16.92 ± 21.52 mL, with a minimum of 0.78 mL and a maximum of 195.15 mL^29^.

### WMH segmentation model

The algorithm for WMH detection in the software comprised a 2D convolutional neural network (CNN) model that processes individual MRI slices sequentially. Initially, pixel values of the input MR images were normalized to fall within the range of 0.0–1.0. Notably, image processing-based bias field corrections were not applied. We made this decision because the effect of the contrast augmentation during training was anticipated to accommodate such variations. Then, the normalized image was resized to 512 × 512 pixels and input into the main CNN model. This CNN model was an ensemble of two U-Net models, each with distinct characteristics. The first U-Net model utilized ResNext50 as the encoder, and the ImageNet pretrained weights were used as the initial weights. The learning rate was set to 0.001, the batch size was set to 15, and the model was trained for 15 epochs using the Adam optimizer. These hyperparameters were obtained using a grid search. During training, the learning rate was decayed using a cosine-annealing learning-rate reducer to facilitate convergence. Matthews correlation coefficient loss was used as the loss function, as originally proposed for skin lesion segmentation^39^. This loss function incorporates a penalty for misclassification of the true negative pixels and is expected to show robust performance. During the training process, data augmentations such as horizontal flips, vertical flips, shifting, rotation, contrast transformation (changing the window width), and grid distortion were applied to all the slices, regardless of WMH presence, to foster the development of a noise-robust model. The second U-Net model used EfficientNet-B5 as the encoder, which was trained in the same manner as the first U-Net. The output of the main CNN model was a threshold of 0.5, and the predicted binary WMH mask was obtained. The number of ensembles was set to two to obtain a balance between processing time and improved accuracy. Ensembles comprising three or more models increased the processing time by approximately 1.5 times or more, although the enhancement in performance was marginal.

Pseudo-labeling was employed to effectively utilize the unlabeled data in the training of the actual model^40^. This technique uses the model’s predictions for unlabeled data to generate labels, which are then used to retrain the model, potentially increasing the diversity of the training dataset and improving generalization performance. First, a model for pseudo-labeling was trained using the PR for training only. The model was then used to predict the WMH regions in unannotated images of 885 participants. These predicted WMH masks can be regarded as pseudo-WMH annotations (pseudo-labeling dataset). The pseudo-labeling model showed a DSC of 0.817 for the PR. Although the pseudo-labeling dataset contains both correct and incorrect predictions, increasing the diversity of the training dataset can be expected to improve the generalization performance. Finally, a new model was trained using both the PR and pseudo-labeling dataset.

### Evaluation metrics

To evaluate WMH segmentation performance, the voxel-wise DSC was used in this study. The DSC is a measure of the agreement between predicted and ground-truth regions and is defined as DSC = 2 × true positive (TP)/(2 × TP + FP + FN), where TP, FP, and FN are the numbers of TP, FP, and FN voxels, respectively. Moreover, recall and precision were calculated using the following formulas: recall = TP/(TP + FN) and precision = TP/(TP + FP). Since there are significantly more voxels in images without versus with WMHs, resulting in a notably higher number of true negatives versus FPs, specificity tends to approach a value near 1. This characteristic makes it unsuitable for detailed comparisons, and thus, was not used as an evaluation metric in this study.

### Processing time measurement

To evaluate the applicability of the model in clinical practice with limited resources, processing time was measured without using a graphics processing unit (GPU). The average of 10 measurements was computed for thick-slice MR images. To facilitate comparison with a previous study^32^, the measured value was corrected for differences in the number of slices and central processing unit (CPU) clock frequencies as follows. In the prior study, time measurements were obtained using MRI with 192 slices on a computer with a 3.5-GHz CPU, whereas our study used 22 slices on a computer without a GPU, equipped with an Intel Core i5-10500 T processor (Intel Corp., Santa Clara, CA, USA) running at 2.30 GHz with 16 GB of memory. Assuming that processing time decreases proportionally with the CPU clock frequency, the corrected processing time, intended for comparison with the results of the previous study, was calculated as follows: corrected processing time = measured time (min) × (192/22) × (2.3/3.5).

### Supplementary Information

Supplementary Table S1.

## Data Availability

Anonymized data from the present study may be shared by the corresponding author upon request from a qualified researcher and upon permission from the institutional review board.

## Abbreviations

2D: Two-dimensional
3D: Three-dimensional
AI: Artificial intelligence
CNN: Convolutional neural network
CPU: Central processing unit
DSC: Dice similarity coefficient
DSWMH: Deep and subcortical white matter hyperintensity
EPVS: Enlarged perivascular space
FLAIR: Fluid-attenuated inversion recovery
FN: False negative
FP: False positive
GPU: Graphics processing unit
MRI: Magnetic resonance imaging
PR: Private Dataset
PVH: Periventricular hyperintensity
T1WI: T1-weighted image
T2WI: T2-weighted image
TP: True positive
WMH: White matter hyperintensity
WMHC: WMH Segmentation Challenge

## References

[1] Wardlaw JM (2013). Neuroimaging standards for research into small vessel disease and its contribution to ageing and neurodegeneration. Lancet Neurol..

[2] van Dijk EJ (2008). Progression of cerebral small vessel disease in relation to risk factors and cognitive consequences: Rotterdam Scan study. Stroke.

[3] Mosley TH (2005). Cerebral MRI findings and cognitive functioning: the Atherosclerosis Risk in Communities study. Neurology.

[4] Doddy RS, Massman PJ, Mawad M, Nance M (1998). Cognitive consequences of subcortical magnetic resonance imaging changes in Alzheimer’s disease: Comparison to small vessel ischemic vascular dementia. Neuropsychiatry Neuropsychol. Behav. Neurol..

[5] O’Brien J (1998). Severe deep white matter lesions and outcome in elderly patients with major depressive disorder: Follow up study. BMJ.

[6] Ter Telgte A (2018). Cerebral small vessel disease: From a focal to a global perspective. Nat. Rev. Neurol..

[7] Prins ND, Scheltens P (2015). White matter hyperintensities, cognitive impairment and dementia: an update. Nat. Rev. Neurol..

[8] Simoni M (2012). Age- and sex-specific rates of leukoaraiosis in TIA and stroke patients: population-based study. Neurology.

[9] Yamasaki T (2021). Prevalence and risk factors for brain white matter changes in young and middle-aged participants with Brain Dock (brain screening): A registry database study and literature review. Aging (Albany NY).

[10] Breteler MM (1994). Cognitive correlates of ventricular enlargement and cerebral white matter lesions on magnetic resonance imaging. The Rotterdam Study. Stroke.

[11] New Guidelines Development Committee for Brain Dock. [The Guideline for Brain Dock 2019]: Kyobunsha; 2019.

[12] Morita A (2019). Value of brain dock (brain screening) system in Japan. World Neurosurg..

[13] Saito I (2006). The guideline for brain dock 2003. Nihon Rinsho.

[14] Zhu W (2022). Automatic segmentation of white matter hyperintensities in routine clinical brain MRI by 2D VB-Net: A large-scale study. Front. Aging Neurosci..

[15] Joo L (2022). Diagnostic performance of deep learning-based automatic white matter hyperintensity segmentation for classification of the Fazekas scale and differentiation of subcortical vascular dementia. PLoS One.

[16] Zijdenbos AP, Forghani R, Evans AC (2002). Automatic, "pipeline" analysis of 3-D MRI data for clinical trials: Application to multiple sclerosis. IEEE Trans. Med. Imaging.

[17] Grimaud J (1996). Quantification of MRI lesion load in multiple sclerosis: A comparison of three computer-assisted techniques. Magn. Reson. Imaging.

[18] Røvang MS (2023). Segmenting white matter hyperintensities on isotropic three-dimensional fluid attenuated inversion recovery magnetic resonance images: Assessing deep learning tools on a Norwegian imaging database. PLoS One.

[19] Ding Y (2020). Using deep convolutional neural networks for neonatal brain image segmentation. Front. Neurosci..

[20] Ding T (2020). An improved algorithm of white matter hyperintensity detection in elderly adults. Neuroimage Clin..

[21] Park G, Hong J, Duffy BA, Lee JM, Kim H (2021). White matter hyperintensities segmentation using the ensemble U-Net with multi-scale highlighting foregrounds. Neuroimage.

[22] Liu L, Kurgan L, Wu FX, Wang J (2020). Attention convolutional neural network for accurate segmentation and quantification of lesions in ischemic stroke disease. Med. Image Anal..

[23] Le M (2019). FLAIR^2^ improves LesionTOADS automatic segmentation of multiple sclerosis lesions in non-homogenized, multi-center, 2D clinical magnetic resonance images. Neuroimage Clin..

[24] Heinen R (2019). Performance of five automated white matter hyperintensity segmentation methods in a multicenter dataset. Sci. Rep..

[25] Zhang Y (2022). A deep learning algorithm for white matter hyperintensity lesion detection and segmentation. Neuroradiology.

[26] Park BY (2018). DEWS (DEep White matter hyperintensity Segmentation framework): A fully automated pipeline for detecting small deep white matter hyperintensities in migraineurs. Neuroimage Clin..

[27] Moeskops P (2018). Evaluation of a deep learning approach for the segmentation of brain tissues and white matter hyperintensities of presumed vascular origin in MRI. Neuroimage Clin..

[28] Gibson E, Gao F, Black SE, Lobaugh NJ (2010). Automatic segmentation of white matter hyperintensities in the elderly using FLAIR images at 3T. J. Magn. Reson. Imaging.

[29] Kuijf HJ (2019). Standardized assessment of automatic segmentation of white matter hyperintensities and results of the WMH Segmentation Challenge. IEEE Trans. Med. Imaging.

[30] Li H (2018). Fully convolutional network ensembles for white matter hyperintensities segmentation in MR images. Neuroimage.

[31] Iwamura M (2024). Thin-slice two-dimensional T2-weighted imaging with deep learning-based reconstruction: Improved lesion detection in the brain of patients with multiple sclerosis. Magn. Reson. Med. Sci..

[32] Rachmadi MF, Valdés-Hernández MDC, Agan MLF, Di Perri C, Komura T (2018). Segmentation of white matter hyperintensities using convolutional neural networks with global spatial information in routine clinical brain MRI with none or mild vascular pathology. Comput. Med. Imaging Graph.

[33] Tran P (2022). Automatic segmentation of white matter hyperintensities: Validation and comparison with state-of-the-art methods on both Multiple Sclerosis and elderly subjects. Neuroimage Clin..

[34] Fiford CM (2020). Automated white matter hyperintensity segmentation using Bayesian model selection: assessment and correlations with cognitive change. Neuroinformatics.

[35] Wu J, Zhang Y, Tang X (2019). Simultaneous tissue classification and lateral ventricle segmentation via a 2D U-net driven by a 3D fully convolutional neural network. Annu. Int. Conf. IEEE Eng. Med. Biol. Soc..

[36] Wu D (2019). Multi-atlas based detection and localization (MADL) for location-dependent quantification of white matter hyperintensities. Neuroimage Clin..

[37] Ling Y, Jouvent E, Cousyn L, Chabriat H, De Guio F (2018). Validation and optimization of BIANCA for the segmentation of extensive white matter hyperintensities. Neuroinformatics.

[38] Manjón JV (2018). MRI white matter lesion segmentation using an ensemble of neural networks and overcomplete patch-based voting. Comput. Med. Imaging Graph.

[39] Abhishek, K. & Hamarneh, G. Matthews correlation coefficient loss for deep convolutional networks: Application to skin lesion segmentation. in *2021 IEEE 18th International Symposium on Biomedical Imaging (ISBI)*. 10.1109/ISBI48211.2021.9433782 (2021).

[40] Lee DH. Pseudo-Label: The simple and efficient semi-supervised learning method for deep neural networks. ICML 2013 Workshop: Challenges in Representation Learning (WREPL) https://www.researchgate.net/publication/280581078/ (2013).

