# Supplementary material for: Artificial intelligence for volumetric measurement of cerebral white matter hyperintensities on thick-slice fluid-attenuated inversion recovery (FLAIR) magnetic resonance images from multiple centers
Source: Sci Rep. 2024 May 2;14:10104. doi: 10.1038/s41598-024-60789-x (PMC11065995; doi:10.1038/s41598-024-60789-x)
Supplement: Supplementary file 1 — Supplementary Table S1. [file 41598_2024_60789_MOESM1_ESM.docx]

**Artificial intelligence for volumetric measurement of cerebral white matter hyperintensities on** **thick-slice fluid-attenuated inversion recovery (FLAIR) magnetic resonance images from multiple centers**

Masashi Kuwabara, Fusao Ikawa, Shinji Nakazawa, Saori Koshino, Daizo Ishii, Hiroshi Kondo, Takeshi Hara, Yuyo Maeda, Ryo Sato, Taiki Kaneko, Shiyuki Maeyama, Yuki Shimahara, and Nobutaka Horie

| Supplementary Table S1. MRI parameters of the Private Dataset with 207 annotated participants | | | | | | | | | |  |  |
| --- | --- | --- | --- | --- | --- | --- | --- | --- | --- | --- | --- |
| Vendor | Scanner type ID^a^ | Scanner | Magnetic field strength (T) | Repetition time (ms) | Echo time (s) | Field of view (mm) | Acquisition matrix | Slice thickness (mm) | Spacing between slices (mm) | Inversion Time (ms) | Number of subjects |
| GE Healthcare | 1 | DISCOVERY MR750w | 3 | 8900 | 140–149 | 220 | 320 × 192 | 6 | 7 | 2040–2050 | 4 |
| GE Healthcare | 1 | DISCOVERY MR750w | 3 | 11000 | 140–149 | 220 | 320 × 192 | 6 | 7 | 2250 | 28 |
| GE Healthcare | 2 | Optima MR450w | 1.5 | 9000 | 120–129 | 240 | 224 × 224 | 5 | 6 | 2358 | 2 |
| GE Healthcare | 3 | SIGNA Architect | 3 | 11000 | 100–109 | 220 | 288 × 224 | 5 | 6 | 2649 | 6 |
| GE Healthcare | 3 | SIGNA Architect | 3 | 11000 | 90–99 | 220 | 288 × 224 | 5 | 6 | 2649 | 1 |
| GE Healthcare | 4 | SIGNA EXCITE | 1.5 | 8000 | 140–149 | 239 | 288 × 224 | 6 | 8 | 2000 | 4 |
| GE Healthcare | 4 | SIGNA EXCITE | 1.5 | 8000 | 140–149 | 240 | 288 × 224 | 6 | 8 | 2000 | 2 |
| GE Healthcare | 5 | Signa HDxt | 1.5 | 9000 | 110–119 | 220 | 320 × 320 | 6 | 7 | 2200 | 1 |
| GE Healthcare | 5 | Signa HDxt | 1.5 | 8000 | 140–149 | 240 | 288 × 224 | 5 | 6.5 | 2000 | 1 |
| GE Healthcare | 5 | Signa HDxt | 1.5 | 8000 | 140–149 | 240 | 288 × 224 | 6 | 8 | 2000 | 1 |
| GE Healthcare | 5 | Signa HDxt | 1.5 | 8920 | 140–149 | 220 | 224 × 192 | 6 | 7 | 2250 | 1 |
| GE Healthcare | 5 | Signa HDxt | 1.5 | 10000 | 140–149 | 220 | 224 × 192 | 6 | 7 | 2250 | 36 |
| GE Healthcare | 6 | Signa HDxt | 3 | 11000 | 140–149 | 220 | 288 × 192 | 5 | 6 | 2250 | 1 |
| GE Healthcare | 6 | Signa HDxt | 3 | 11000 | 140–149 | 220 | 288 × 224 | 5 | 6 | 2250 | 16 |
| Philips | 7 | Ingenia | 1.5 | 10000 | 100–109 | 230 | 320 × 198 | 5 | 6 | 2500 | 1 |
| Philips | 7 | Ingenia | 1.5 | 10000 | 110–119 | 229 | 268 × 246 | 5 | 6 | 2700 | 27 |
| Philips | 7 | Ingenia | 1.5 | 10000 | 120–129 | 229 | 320 × 223 | 5 | 6 | 2500 | 1 |
| Philips | 7 | Ingenia | 1.5 | 10000 | 120–129 | 230 | 320 × 228 | 5 | 6 | 2500 | 29 |
| Philips | 7 | Ingenia | 1.5 | 10000 | 120–129 | 230 | 320 × 234 | 5 | 6 | 2500 | 2 |
| Philips | 7 | Ingenia | 1.5 | 10000 | 120–129 | 230 | 320 × 242 | 5 | 6 | 2500 | 1 |
| Siemens Healthineers | 8 | Avanto | 1.5 | 9000 | 100–109 | 221 | 256 × 177 | 5 | 6 | 2500 | 20 |
| Siemens Healthineers | 9 | Symphony | 1.5 | 9000 | 100–109 | 209 | 320 × 211 | 5 | 6 | 2500 | 14 |
| Siemens Healthineers | 9 | Symphony | 1.5 | 9000 | 100–109 | 209 | 320 × 211 | 5 | 6.25 | 2500 | 3 |
| Siemens Healthineers | 9 | Symphony | 1.5 | 9000 | 100–109 | 218 | 320 × 230 | 5 | 6 | 2500 | 5 |
| ^a^Scanner type ID: a unique ID for each combination of scanner and magnetic field strength.  MRI, magnetic resonance imaging; T, Tesla | | | | |  |  |  |  |  |  |  |
